# Adaptive evolution of extensive drug resistance and persistence in epidemic ST11 KPC-producing *Klebsiella pneumoniae* during antimicrobial chemotherapy

**DOI:** 10.1128/aac.01235-24

**Published:** 2024-12-10

**Authors:** Tinghua Li, Yiwei Zhu, Guoxiu Xiang, Ziyang Xu, Haihui Yang, Min Li, Zhen Shen

**Affiliations:** 1 Department of Laboratory Medicine, Renji Hospital, School of Medicine, Shanghai Jiao Tong University 569077 , Shanghai, China; 2 Department of Critical Care Medicine, Renji Hospital, School of Medicine, Shanghai Jiao Tong University 659335 , Shanghai, China; 3 Department of Laboratory Medicine, Shanghai YangZhi Rehabilitation Hospital (Shanghai Sunshine Rehabilitation Center), School of Medicine, Tongji University 12476 , Shanghai, China; Universita degli studi di roma La Sapienza, Rome, Italy

**Keywords:** KPC-producing *Klebsiella pneumoniae*, MgrB, colistin resistance, virulence, bacterial persistence

## Abstract

The global rise of carbapenem-resistant *Klebsiella pneumoniae*, including strains producing *K. pneumoniae* carbapenemase (KPC) types, poses a significant public health challenge due to their resistance to critical antibiotics. Treatment options for infections caused by KPC-producing *K. pneumoniae* (KPC-KP) are increasingly limited, particularly as these strains develop resistance to last-line antibiotics such as ceftazidime/avibactam and colistin. This study investigates the evolution of antibiotic resistance and persistence in a series of clonally related ST11 KPC-KP strains isolated from a single patient undergoing extended antimicrobial treatment. The patient, a 47-year-old male with a history of kidney transplantation, developed multiple KPC-KP lung infections during his hospital stay. Resistance to colistin and ceftazidime/avibactam emerged during treatment with these antibiotics. Key resistance mechanisms identified included the integration of IS*Kpn26* into *mgrB* gene, leading to *mgrB* inactivation and colistin resistance, and the emergence of novel *bla*_KPC-2_ variants (*bla*_KPC-71_ and *bla*_KPC-179_) that confer resistance to ceftazidime/avibactam. Despite the development of colistin resistance in a ceftazidime/avibactam-resistant KPC-KP strain following combination therapy, the patient’s clinical condition significantly improved. Phenotypic assays showed that *mgrB* disruption in KPC-KP resulted in increased biofilm formation and higher susceptibility to phagocytosis. In mouse models, KPC-KP strains with *mgrB* disruption showed reduced virulence, increased lung colonization and persistence, and a lower inflammatory response, suggesting that *mgrB* disruption facilitates the transition from acute infection to colonization. This study highlights the complex interplay between antibiotic resistance and bacterial fitness, offering insights into why some patients experience clinical improvement despite severe drug resistance and incomplete bacterial clearance.

## INTRODUCTION

The global spread of carbapenem-resistant *Klebsiella pneumoniae* (CRKP) presents a significant challenge to public health due to its resistance to commonly used antibiotics. The primary cause of carbapenem resistance is the production of carbapenemases, including the *K. pneumoniae* carbapenemase (KPC) types. In China, approximately 80% of CRKP clinical isolates produce KPC, and ST11 is the dominant KPC-producing *K. pneumoniae* (KPC-KP) clone (1, 2). Treatment options for KPC-KP infections are limited, primarily relying on last-line antibiotics, such as ceftazidime/avibactam and colistin. However, resistance to these antibiotics in KPC-KP has been increasingly reported, raising concerns due to the scarcity of alternative treatments (3, 4).

The emergence of novel KPC variants is the dominant mechanism that leads to ceftazidime/avibactam resistance in KPC-KP (5). The KPC variant usually refers to the substitution, insertion, or deletion of one or more amino acids compared to wild KPC types (such as KPC-2 and KPC-3). Over 150 KPC variants have been identified globally, most discovered in the past 3 years (5). These variants modify the KPC structure, enhancing its affinity for ceftazidime and reducing its binding to avibactam, thereby mediating resistance to ceftazidime/avibactam (6).

Colistin resistance in *K. pneumoniae* involves modifications to the lipid A component of lipopolysaccharide (LPS), driven by mutations in the two-component systems (TCSs) PmrAB, PhoPQ, or CrrAB, or by acquiring a plasmid with the *mcr-1* gene (7, 8). Despite the contribution of multiple TCSs in lipid A modification, most colistin resistance arises from mutations that inactivate the MgrB protein, which negatively regulates PhoPQ (9, 10). The inactivation effect may be due to the insertion of an insertion sequence or a mutation that results in a premature stop codon. Consequently, PhoPQ and PmrAB are overactivated when MgrB is inactivated, increasing the lipid A modifications that confer colistin resistance (11).

LPS is one of the major pathogen-associated molecular patterns involved in the activation of the host’s innate immune system in the early stages of infection, and its alterations dampen immune recognition (12, 13). Beyond antimicrobial resistance, modifications to LPS have the potential to alter the host inflammatory response, which may manifest as altered bacterial growth and virulence (14, 15). However, the impact of *mgrB* disruption on the virulence of *K. pneumoniae* remains debated (16–18). The inconsistent data on biological cost can be attributed to the type of mutation, the strain tested, and the type of assays conducted. This is particularly critical given the increasing number of *K. pneumoniae* infections caused by virulent clones and the ease with which *mgrB* mutations arise in the hospital setting.

To address the clinically relevant issues, a series of clonally related KPC-KP strains were identified from a single patient. Through whole-genome sequencing and bioinformatic analysis, we traced the dynamic evolution of KPC-KP resistance to ceftazidime/avibactam and colistin during extended antimicrobial therapy. Key resistance mechanisms identified included the integration of insertion sequence IS*Kpn26* into the *mgrB* gene, leading to *mgrB* inactivation and colistin resistance, and the emergence of novel *bla*_KPC-2_ variants (*bla*_KPC-71_ and *bla*_KPC-179_) that confer resistance to ceftazidime/avibactam. Despite the development of colistin resistance in a ceftazidime/avibactam-resistant KPC-KP strain following combination therapy, the patient’s clinical condition significantly improved. We investigated the impact of colistin resistance on the physiological fitness and virulence of KPC-KP using *in vitro* assays and mouse infection models. Our study demonstrates that KPC-KP with *mgrB* disruption shows increased susceptibility to phagocytosis, an attenuated inflammatory response, and reduced virulence *in vivo*, facilitating the transition from acute infection to colonization and persistence in the respiratory tract.

## MATERIALS AND METHODS

### Medical history of the clinical case

**Fig 1:**
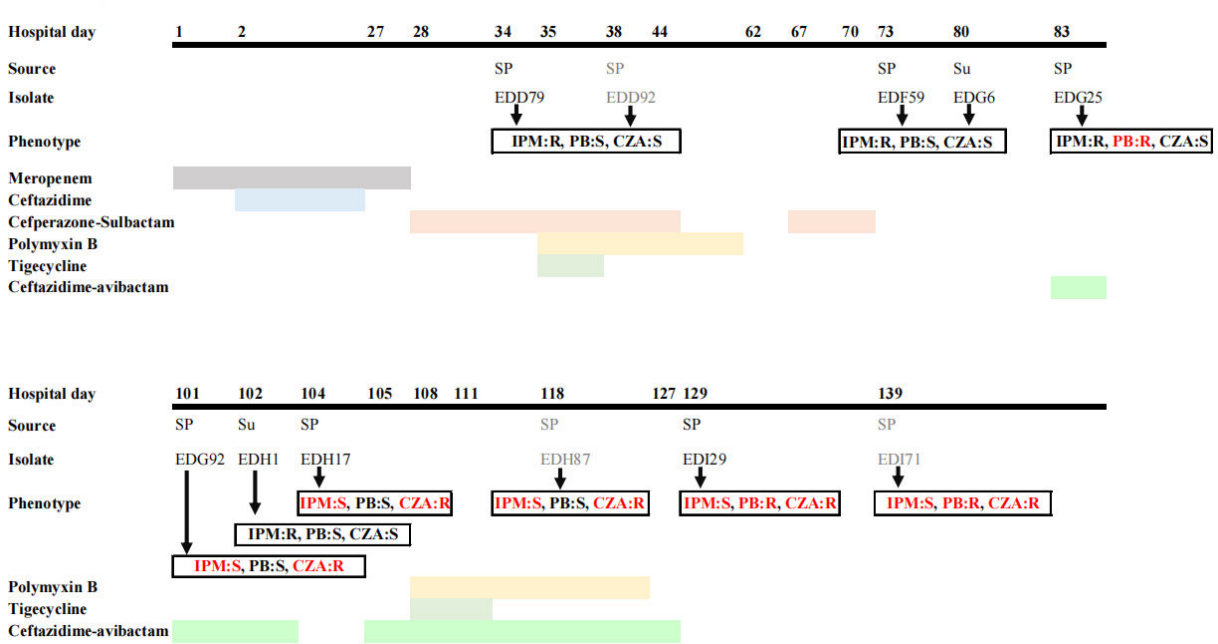
Time courses of infection and treatment of the patient with KPC-KP infection. Three strains, EDD92, EDH87, and EDI71, were excluded from whole-genome sequencing analysis due to their antimicrobial resistance profiles resembling those of previously isolated strains. The color blocks represent the treatment periods for the corresponding antibiotics listed in each row. SP, sputum; Su, peritoneal fluid; IPM, imipenem; PB, polymyxin B; CZA, ceftazidime/avibactam; S, susceptible; R, resistant.

A 47-year-old male patient was admitted to the department of urology due to uremia and subsequently underwent kidney transplantation. The patient then got severe acute respiratory syndrome coronavirus 2 infection, leading to respiratory failure. In response to persistent declines in blood oxygen saturation, he received endotracheal intubation and mechanical ventilation. During treatment, the first KPC-KP strain, EDD79, was isolated from his sputum. The patient was then treated with polymyxin B, tigecycline, and cefoperazone-sulbactam, replacing the initial regimen of meropenem and ceftazidime (Fig. 1).

The patient’s pneumonia was effectively managed, allowing the discontinuation of polymyxin B and tigecycline. However, after 12 days of polymyxin B withdrawal, the patient’s body temperature rose again, indicating a severe pulmonary infection. Two KPC-KP strains, similar in antimicrobial susceptibility to EDD79, were isolated from sputum and peritoneal fluid. Concerningly, a colistin-resistant KPC-KP strain, EDG25, was identified from the sputum (Fig. 1). The patient was then treated with ceftazidime/avibactam monotherapy. Despite this, recurrent pneumonia occurred, and a ceftazidime/avibactam-resistant, colistin-susceptible KPC-KP strain, EDG92, was isolated from the sputum (Fig. 1).

The patient received combination therapy with ceftazidime/avibactam and polymyxin B; however, a KPC-KP strain, EDI29, exhibiting resistance to both ceftazidime/avibactam and polymyxin B, was identified in the sputum following 20 days of treatment (Fig. 1). Despite the emergence of this extensive drug-resistant strain, the patient’s pulmonary symptoms improved, and his vital signs stabilized. However, his kidney transplant ultimately failed due to the prolonged post-transplant infection. Consequently, the patient was transferred to a rehabilitation facility for further management.

### Antimicrobial susceptibility testing

Clinical isolates were identified by matrix-assisted laser desorption ionization-time of flight mass spectrometry (Autobio, Henan, China). Antimicrobial susceptibility testing was performed with the reference broth microdilution method. The results were interpreted according to the CLSI 2023 guideline, except for tigecycline and polymyxin, where the breakpoint was defined by the EUCAST, version 12.0 (http://www.eucast.org/). Carbapenemases were detected with the colloidal gold immunoassay, and the carbapenemase genes were confirmed with the Xpert Carba-R assay (Cepheid, Sunnyvale, USA).

### Genomic sequencing and analysis

Genomic DNAs of KPC-KP isolates were extracted and subjected to whole-genome sequencing using Illumina NovaSeq 6000 platform (Illumina, San Diego, USA). Three strains, EDD92, EDH87, and EDI71, were excluded from whole-genome sequencing analysis due to their antimicrobial resistance profiles resembling those of previously isolated strains. The extensive drug-resistant strain, EDI29, was selected for long-read PacBio RS sequencing on Sequel II System. The raw reads were trimmed and filtered to remove low-quality sequences and adaptors by Trimmomatic (version 0.36). Antimicrobial resistance genes and plasmid replicon analysis were performed using ResFinder and PlasmidFinder tools via the CGE server (19). Multilocus sequence typing and capsular (K) type were determined using Kleborate 1.0.0. Pan-genome analysis was done using Roary version 3.11.2. Core genes are defined as those present in all isolates. Core genome single-nucleotide polymorphisms (cgSNPs) were extracted using SNP-sites from core gene alignment and filtered using VCF tools version 0.1.17. A maximum likelihood phylogenomic tree of these genomes was constructed by RAxML-NG version 1.1.0 (20).

### Cloning of *bla*_KPC-2_ variants

The wild-type *bla*_KPC-2_ and novel variants gene sequences containing the ribosomal binding site and the upstream promoter were amplified from the corresponding KPC-KP strains. The PCR products were purified and then cloned into the pHSG398 vector. The recombinant plasmids were introduced into the *Escherichia coli* DH5α strain via chemical transformation. Luria-Bertani (LB) agar plates containing 20 µg/mL chloramphenicol were used to select transformants, which were further verified by PCR and sequencing.

### Construction of *mgrB* deletion mutant of *K. pneumoniae* EDH17

To construct markerless mutants, we generated vectors for each mutation using pCONJ5H, in which the hygromycin phosphotransferase gene (*hph*) was employed to replace the resistance genes in pCONJ working vectors. The upstream and downstream flanking regions (approximately 500 bp) of *mgrB* were amplified by PCR with the relevant primers and assembled into pCONJ5H with NEBuilder HiFi DNA Assembly Cloning Kit (New England Biolabs). The *mgrB* deletion mutant of *K. pneumoniae* EDH17 was then generated by conjugation as previously described (21).

### Quantitative real-time polymerase chain reaction

Total RNA from the mid-logarithmic phase culture of EDH17, EDI29, and EDH17Δ*mgrB* was extracted with the RNeasy Kit (Qiagen) according to the manufacturer’s instructions, checked for purity, and concentration was assessed using a NanoDrop spectrophotometer (Thermo Scientific). A total of 0.5 mg RNA was reverse-transcribed to complementary DNA. Real-time PCR was performed on a 7500 real-time PCR system (Applied Biosystems) with primers as previously reported (22). Gene expression was calculated using the 2^−ΔΔCT^ method.

### Bacterial growth

To construct an *in vitro* growth curve, we cultured three KPC-KP strains, EDH17, EDI29, and EDH17Δ*mgrB*, in LB broth. The strains were grown overnight in LB at 37°C with constant shaking. The overnight culture was then diluted 1:1,000 in 5 mL of fresh LB and incubated at 37°C with shaking. We measured bacterial growth by recording the optical density at 600 nm at 0, 1, 2, 4, 6, and 8 hours post-dilution.

### Serum resistance assay

The growth of *K. pneumoniae* strain in human serum was determined as previously described (23). Briefly, bacterial suspension containing 1 × 10^6^ cell forming unit (CFU)/mL was collected from exponential phase cultures, followed by mixing with pooled human serum at a ratio of 1:3 and incubated at 37°C with constant shaking. CFU counts were determined for the initial mixture, as well as that after 1, 2, and 3 hours of incubation.

### Neutrophil phagocytosis assay

Neutrophils were isolated from the venous blood of healthy volunteers using Ficoll-Hypaque gradient density centrifugation. To measure neutrophil phagocytosis activity, neutrophils (10^6^ cells) were mixed with *K. pneumoniae* (10^7^ CFU) in a final volume of 500 µL (23). The mixture was incubated at 37°C for 15, 30, and 60 minutes with gentle rotation. After incubation, the mixture was washed three times with phosphate-buffered saline (PBS) and treated with 200 µg/mL hygromycin in growth medium to kill extracellular bacteria. The cells were then washed three more times and lysed with 0.1% Triton-X100 for 20 minutes. Serial dilutions of the lysate were plated on LB agar to determine CFUs.

### Adhesion of *K. pneumoniae* to human alveolar epithelial cells A549

Human alveolar epithelial cells (A549) were cultured in Dulbecco's modified Eagle medium (DMEM) with 10% fetal bovine serum at 37°C and 5% CO_2_. Bacteria were grown to the mid-logarithmic phase and washed twice with DMEM. A549 cells and bacteria were mixed at a 1:10 ratio (multiplicity of infection [MOI] = 10) and incubated for 2 hours (24). After incubation, the culture supernatants were discarded, and the cells were washed three times with sterile PBS to remove non-adherent bacteria. A549 cells were lysed with 0.1% Triton solution. The number of adhering bacteria (CFU) was determined by serially diluting the cell lysates and plating them onto LB agar plates.

### Biofilm formation

Biofilm production was determined as previously described (25). Overnight cultures were diluted 1:1,000 in LB medium, and 200 µL of this dilution was transferred to wells of untreated 96-well polystyrene plates. After a 24-hour incubation at 37°C, the wells were washed three times with sterile PBS, and 100 µL of 0.1% crystal violet was added. After a 10-minute incubation, the crystal violet was removed, and the excess stain was washed off with running water. Biofilm formation was quantified by measuring the absorbance at 570 nm.

### Mice infection model

Pathogen-free, 6-week-old to 8-week-old female BALB/c mice were used for the infection models. *K. pneumoniae* strains EDH17 and EDI29, along with the corresponding *mgrB* deletion mutant of EDH17, were grown to mid-logarithmic phase, washed, and resuspended in sterile PBS. Each mouse was injected intraperitoneally with 100 µL of bacterial suspension or PBS as a control (23). To analyze bacterial loads, mice challenged with 5 × 10^6^ CFU were euthanized after 48 hours to collect liver and spleen samples. Serially diluted homogenates of these organs were plated for bacterial quantification. Representative tissue samples were also subjected to hematoxylin and eosin staining.

For the lung colonization model, mice were infected intranasally with a fresh mid-log phase dose of 5 × 10^7^ CFU *K*. *pneumoniae* strain in 20 µL PBS (26). Mice were euthanized at predetermined times post-infection (days 1, 3, 5, and 7), and the lungs were removed for assessment of bacterial colonization and growth. Bacterial counts were derived from four mice per time point.

### Cytokine detection

Bronchoalveolar lavage fluid (BALF) was collected 72 hours post-infection. Each mouse was euthanized by deep ether anesthesia, and the trachea was exposed. A polyethylene catheter was inserted into the trachea, and bronchoalveolar lavage was performed using a 1 mL syringe loaded with 0.8 mL of PBS (26). The PBS was slowly injected and aspirated three times. The recovered lavage fluid was collected in 1.5 mL EP tubes and kept on ice. Cytokine levels in BALF were measured using an ELISA Kit (R&D Systems).

### Statistical analysis

Statistical analysis was performed using Graph Pad Prism version 8.0. Comparisons of two groups were performed using unpaired, two-tailed *t*-tests or Mann-Whitney tests, depending on Shapiro-Wilk normal distribution tests. Analyses of three or more groups were performed using one-way or two-way analyses of variance, as appropriate, with Tukey’s post-tests. QQ plots showed only minimal deviation from normal distribution, which is why analyses of variance were generally used for consistency. All error bars show the standard deviation (SD).

## RESULTS

### Disruption of *mgrB* and *bla*_KPC-2_ substitutions contributed to colistin and ceftazidime/avibactam resistance

**Fig 2:**
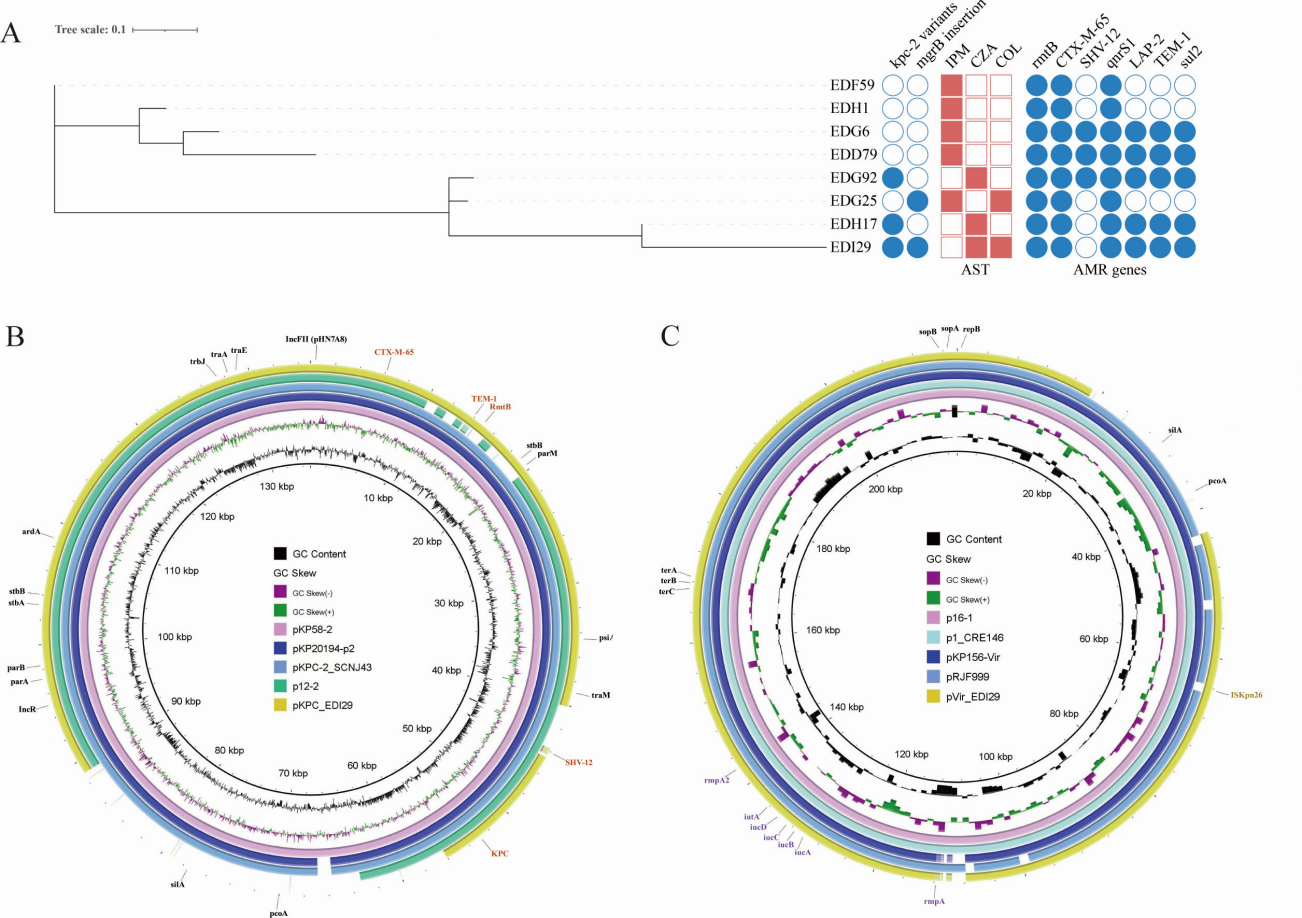
Phylogeny of KPC-KP strains and plasmid structures. (**A**) Phylogeny of KPC-KP strains based on cgSNPs. AST, antimicrobial susceptibility testing; AMR genes, antimicrobial resistance genes. The filled block of color indicates the presence of antimicrobial resistance genes and resistance to the corresponding antibiotics. (**B and C**) Comparative analysis of pKPC-EDI29 and pVir-EDI29 with other reference plasmids. The circular map was created with the BLAST Ring Image Generator. The reference plasmid sequences were obtained from GenBank and listed with plasmid names.

**Fig 3:**
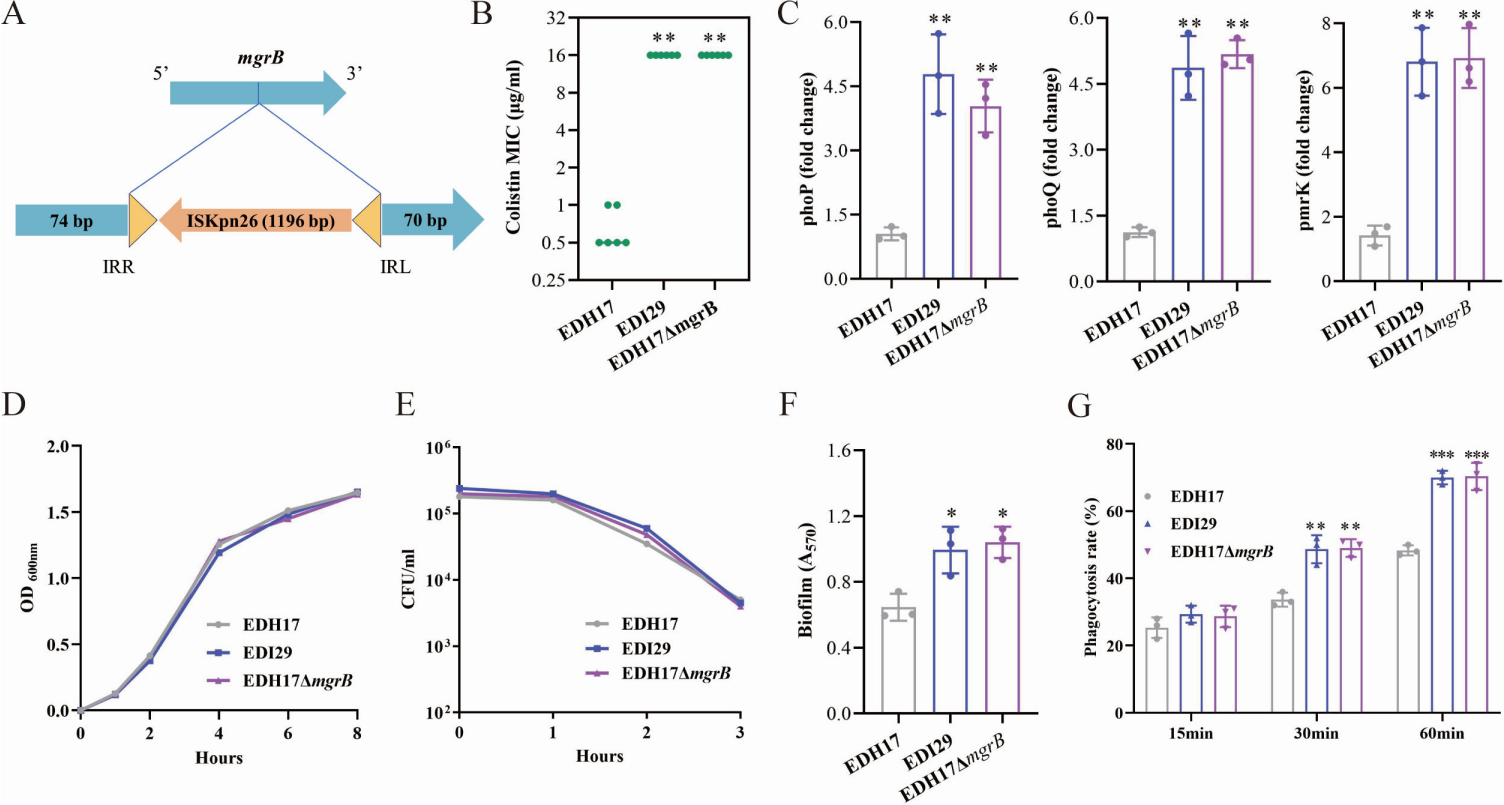
Phenotypic changes of KPC-KP with *mgrB* disruption. (**A**) Schematic depiction of the insertion event observed within the *mgrB* gene. The left and right inverted repeats are denoted as IRR and IRL, respectively. (**B**) Colistin MIC values of *K. pneumoniae* EDH17, EDI29, and *mgrB* deletion mutant of EDH17 (EDH17Δ*mgrB*). (**C**) Gene expression of *phoP*, *phoQ*, and *pmrK*. (**D**) Growth curves. (**E**) Serum resistance assay. (**F**) Biofilm formation. (**G**) Neutrophil phagocytosis assay. Asterisks denote statistically significant differences compared to EDH17. *P < 0.05, **P < 0.01, ***P < 0.001.

Whole-genome sequencing unveiled the clonal relationship among these KPC-KP strains, and all strains belonged to ST 11 (Fig. 2A). cgSNP analysis indicated that isolates EDG25 (colistin-resistant), EDG92 and EDH17 (ceftazidime/avibactam-resistant), and EDI29 (colistin and ceftazidime/avibactam-resistant) were likely direct derivatives of the initial isolate, EDD79, with SNPs ranging from 6 to 13 compared to EDD79 (Table S1). The colistin resistance in *K. pneumoniae* strains EDG25 and EDI29 was attributed to the integration of the insertion sequence IS*Kpn26* into the *mgrB* gene. Both strains exhibited insertions at position +74, leading to *mgrB* inactivation (Fig. 3A). Quantitative reverse transcription-PCR (qRT-PCR) analysis of *phoP* and *phoQ* transcription showed a threefold to fivefold increase in EDI29 compared to EDH17 (Fig. 3C). Additionally, the *pmrK* gene, part of the *pmrHFIJKLM* operon, was activated in the colistin-resistant EDI29 strain (Fig. 3C).

To further characterize the genomic profile of these KPC-KP isolates, we generated the complete genome of the extensively drug-resistant strain EDI29 using a combination of Illumina and long-read PacBio RS sequencing. Two key plasmids were identified: pVir and pKPC. The IncHI1B-type pVir plasmid harbors key virulence genes such as *rmpA*, *rmpA2*, and *iucABCDiutA* (Fig. 2C). The *rmpA2* gene in all sequenced KPC-KP isolates contains a premature stop codon, rendering it non-functional. The pVir plasmid exhibits 86% sequence coverage and 99.41% identity with the canonical virulence plasmid pLVPK (27). The pVir shares a conserved backbone with pLVPK while the FIBK replicon has been deleted. Previous epidemiological studies have shown that pVir is commonly found among ST11 KPC-KP isolates in China (2). The insertion sequence IS*Kpn26* was also identified on pVir in EDI29 (Fig. 2C). The pKPC plasmid shares a conserved backbone with the epidemic IncFII(pHN7A8)/IncR-type KPC plasmid (Fig. 2B). It has primarily spread among ST11 *K. pneumoniae*, particularly in China (2). Except for *bla*_KPC_, several other resistance genes, including *bla*_CTX-M-65_, *bla*_TEM-1_, and *rmtB*, were also identified on pKPC (Fig. 2B).

Two novel *bla*_KPC-2_ variants, *bla*_KPC-71_ and *bla*_KPC-179_, were identified in ceftazidime/avibactam-resistant KPC-KP strains (Table 1). In comparison to *bla*_KPC-2_, *bla*_KPC-71_ displayed a mutated nucleotide and a three-nucleotide insertion at positions 542 to 545, resulting in a serine insertion between Ambler positions 182 and 183 (28). The emergence of *bla*_KPC-179_ was attributed to a single base mutation at G394A, leading to the A132T amino acid mutation in KPC-71. Cloning and expression of *bla*_KPC-71_ and *bla*_KPC-179_ in *E. coli* DH5α demonstrated that both KPC variants could confer resistance to ceftazidime/avibactam while restoring susceptibility to carbapenems (Table 2).

**TABLE 1:** Characteristics of KPC-KP strains identified in the study^*a*^

Strains	EDD79	EDF59	EDG6	EDG25	EDG92	EDH1	EDH17	EDI29
KPC variants	KPC-2	KPC-2	KPC-2	KPC-2	KPC-71	KPC-2	KPC-179	KPC-179
Disruption of *mgrB*	/^*b*^	/	/	+	/	/	/	+
MIC of antimicrobial agents, μg/mL								
Ceftazidime	≥64	≥64	≥64	≥64	≥64	≥64	≥64	≥64
Cefepime	≥64	≥64	≥64	≥64	≥64	≥64	≥64	≥64
Aztreonam	≥64	≥64	≥64	≥64	≥64	≥64	≥64	≥64
Piperacillin/tazobactam	≥128	≥128	≥128	≥128	64	≥128	64	64
Meropenem	≥32	≥32	≥32	≥32	**2**	≥32	**2**	**4**
Imipenem	≥32	≥32	≥32	≥32	**0.25**	≥32	**0.25**	**0.5**
Ceftazidime/avibactam	2	2	2	2	**128**	2	**≥256**	**≥256**
Gentamicin	≥16	≥16	≥16	≥16	≥16	≥16	≥16	≥16
Amikacin	≥64	≥64	≥64	≥64	≥64	≥64	≥64	≥64
Ciprofloxacin	≥4	≥4	≥4	≥4	≥4	≥4	≥4	≥4
Levofloxacin	≥8	≥8	≥8	≥8	≥8	≥8	≥8	≥8
Polymyxin B	0.25	1	1	**16**	0.5	0.5	0.5	**16**
Tigecycline	0.5	0.5	0.25	0.5	0.5	0.5	0.5	0.5

^*a*^ MIC values in bold revealed significant differences when compared with other KPC-KP strains. Three KPC-KP strains, EDD92, EDH87, and EDI71, which have not been subject to whole-genome sequencing, were not listed.

^*b*^ /, strains without *mgrB* disruption.

**TABLE 2:** Susceptibility of *E. coli* DH5α transformant with KPC variants to antimicrobial agents

Antimicrobial agents	MIC of pHSG398 with KPC-2 variants (μg/mL)			
	KPC-2	KPC-71	KPC-179	pHSG398
Ceftazidime	16	≥64	≥64	0.125
Cefepime	8	1	2	0.125
Aztreonam	≥64	2	2	≤0.25
Piperacillin/tazobactam	≥128	16	8	≤1
Imipenem	8	≤0.25	≤0.25	≤0.25
Meropenem	2	≤0.25	≤0.25	≤0.25
Ceftazidime/avibactam	0.25	16	32	0.125
Ciprofloxacin	≤0.25	≤0.25	≤0.25	≤0.25
Levofloxacin	≤0.125	≤0.125	≤0.125	≤0.125
Amikacin	≤1	≤1	≤1	≤1
Tigecycline	≤0.25	≤0.25	≤0.25	≤0.25
Polymyxin B	≤0.25	≤0.25	≤0.25	≤0.25

### Phenotypic changes of KPC-KP with *mgrB* disruption

We assessed the impact of *mgrB* inactivation on colistin resistance by evaluating bacterial growth in both rich media (LB) and human serum. Phylogenetic analysis based on cgSNPs revealed that *K. pneumoniae* EDI29 exhibited the closest genetic relationship with the previously isolated ceftazidime/avibactam-resistant strain EDH17, with only two SNPs identified between them (Table S1). Growth rates of the colistin-resistant strain EDI29 were similar to those of the colistin-susceptible parental strain EDH17, and there were no significant differences between EDH17 and the *mgrB* deletion mutant (Fig. 3D and E). Biofilm formation was examined using a polystyrene microtiter plate assay, which revealed that strain EDI29 had a greater ability to form biofilms compared to EDH17 (Fig. 3F). Additionally, a human neutrophil phagocytosis assay showed that EDI29 was more susceptible to phagocytosis than EDH17 (Fig. 3G). These findings suggest that *mgrB*-dependent colistin resistance in KPC-KP may come with a biological cost, potentially affecting survival fitness.

### Attenuated virulence of KPC-KP with *mgrB* disruption in mice

**Fig 4:**
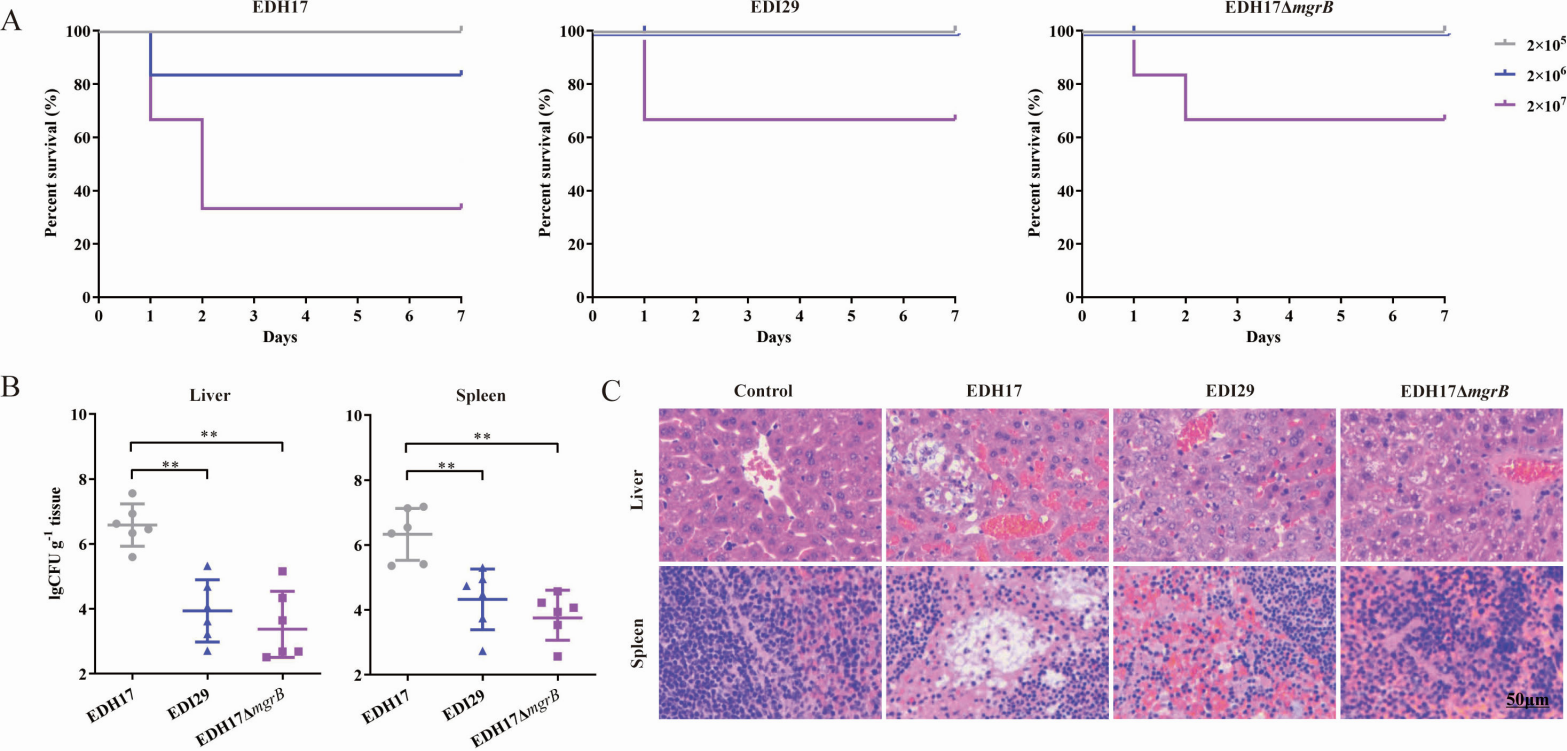
Attenuated virulence of KPC-KP with *mgrB* disruption in mice. (**A**) Survival of mice (*n* = 6 per group) intraperitoneally inoculated with *K. pneumoniae* EDH17, EDI29, and EDH17Δ*mgrB*. (**B**) Bacterial loads in aseptically homogenized liver and spleen of mice euthanized at 48 hours post-infection. Values were presented as mean with SD. Asterisks denote statistically significant differences compared to EDH17. **P < 0.01. (**C**) Representative histopathology of liver and spleen tissues (hematoxylin-eosin staining).

Compared to colistin-resistant EDI29 and EDH17Δ*mgrB*, virulence of the colistin-susceptible parental strain EDH17 was significantly higher, resulting in 66.7% mortality with an inoculum of 2 × 10^7^ CFU at 2 days (Fig. 4A). The liver and spleen of mice at 48 hours post-infection with 5 × 10^6^ CFU showed significantly higher bacterial loads with EDH17 than with EDI29 or EDH17Δ*mgrB* (Fig. 4B). Histopathological analyses of the liver and spleen supported a trend of EDH17 causing more severe damage, with significant congestion in the hepatic lobules and vacuolar degeneration in the spleen, compared with EDI29 or EDH17Δ*mgrB* (Fig. 4C). Additionally, since only six mice were used per condition, further studies with larger animal groups are needed to validate the findings.

### Disruption of *mgrB* in KPC-KP enhances lung colonization and persistence

**Fig 5:**
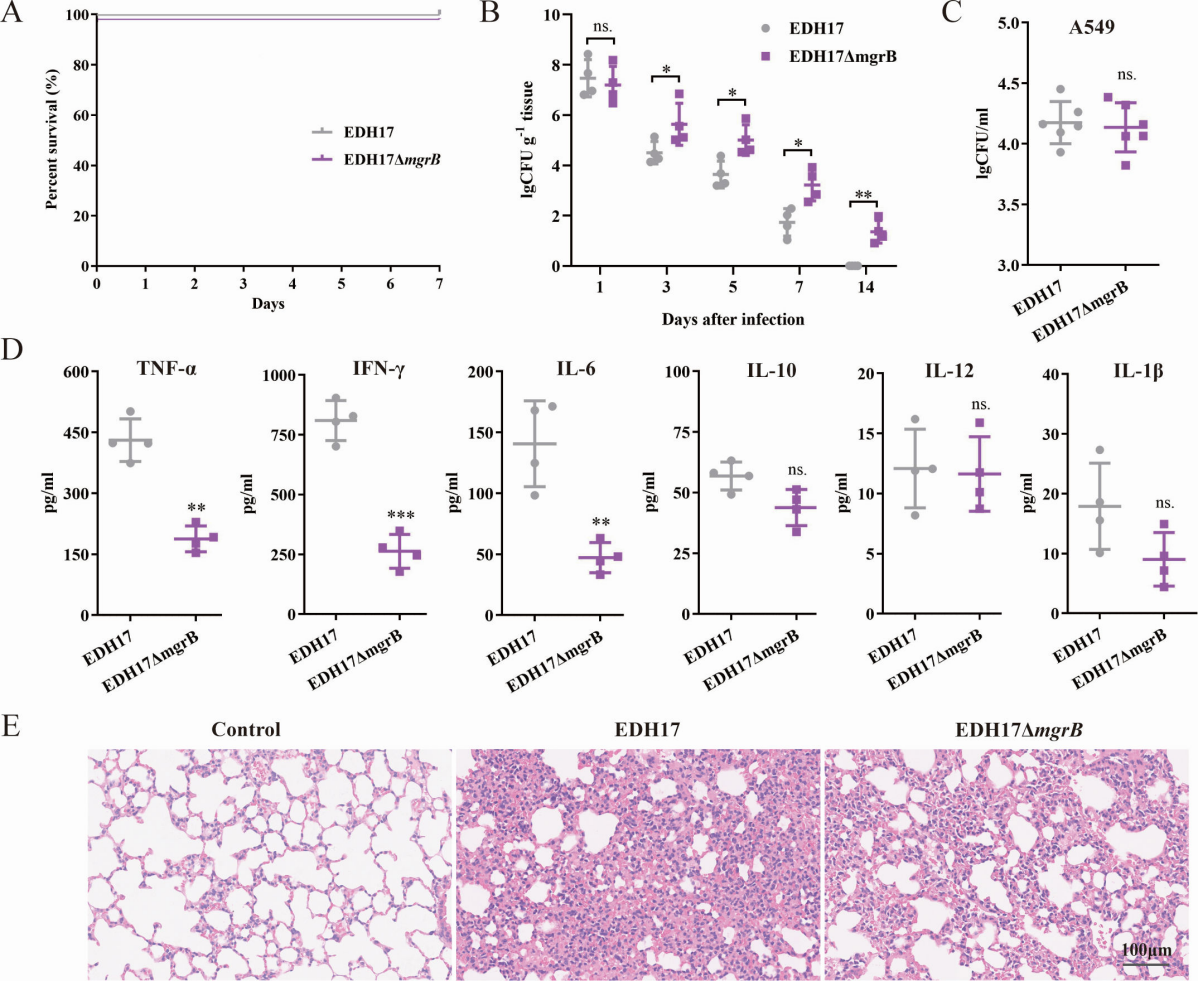
Disruption of *mgrB* in KPC-KP enhances lung colonization and persistence. (**A**) Survival of mice (*n* = 6 per group) intranasally inoculated with 5 × 10^7^ CFU *K*. *pneumoniae* EDH17 and EDH17Δ*mgrB*. (**B**) Mouse lung colonization model with intranasally inoculated with 5 × 10^6^ *K. pneumoniae*. Bacterial loads in the lung post-infection. (**C**) Adhesion of *K. pneumoniae* to human alveolar epithelial cells A549. (**D**) Cytokine production in BALF of mice infected with 5 × 10^6^ CFU *K*. *pneumoniae* for 3 days. (**E**) Representative histopathology of lung tissues (hematoxylin-eosin staining). Asterisks denote statistically significant differences compared to EDH17. *P < 0.05, **P < 0.01, ***P < 0.001, ns, not significant.

To evaluate KPC-KP colonization levels in the mouse lung, mice were inoculated intranasally, and tissue samples were analyzed at various time points to determine bacterial counts. One day post-infection, high bacterial loads were observed in the lungs, with no significant difference in CFUs between mice infected with EDH17 or EDH17Δ*mgrB* (Fig. 5B). After 3 days, bacterial loads decreased in both groups. However, EDH17Δ*mgrB* showed significantly higher colonization compared to EDH17 at all subsequent time points. By day 14, EDH17Δ*mgrB* was still present in the lungs of all tested mice, whereas EDH17 was undetectable (Fig. 5B).

We also measured inflammatory cytokine levels (TNF-α, IFN-γ, IL-1β, IL-6, IL-10, and IL-12) in the BALF supernatant 3 days post-infection. There were statistically significant decreases in TNF-α, IFN-γ, and IL-6 in mice infected with EDH17Δ*mgrB* compared to those infected with EDH17 (Fig. 5D). Histopathological analyses of the lungs supported a trend of EDH17 causing more severe damage in the alveoli, with severe infiltration of lymphocytes and adjacent alveolar hyperplasia in the lung parenchyma, compared with EDH17Δ*mgrB* (Fig. 5E). Adhesion to human alveolar epithelial cells (A549) was similar between EDH17 and EDH17Δ*mgrB* (Fig. 5C). These findings suggest that LPS modification promotes KPC-KP colonization and persistence in the respiratory tract by reducing the inflammatory response.

## DISCUSSION

Colistin and ceftazidime/avibactam are crucial antibiotics commonly used to treat KPC-KP infections. However, in this patient, the use of polymyxin B led to the development of colistin resistance in KPC-KP. Subsequent treatment with ceftazidime/avibactam resulted in the emergence of inhibitor-resistant *bla*_KPC-2_ variants. Under the selective pressure of these antimicrobials, bacteria underwent substantial genomic modifications to introduce advantageous variations. The disruption of *mgrB* by IS*Kpn26* and the mutations in *bla*_KPC-2_ contributed to the convergence of colistin and ceftazidime/avibactam resistance in KPC-KP. The genomic plasticity induced by antibiotic exposure can promote the development of an extensively drug-resistant profile in KPC-KP, thereby constraining already limited treatment options.

Our study highlights the relationship between antimicrobial resistance and virulence in the epidemic ST11 KPC-KP strain. Despite the development of colistin resistance in a ceftazidime/avibactam-resistant KPC-KP strain following combination therapy with these antibiotics, the patient’s clinical condition significantly improved. Clinical indicators suggested that this extensively drug-resistant strain colonized the respiratory tract rather than causing an active infection. In the mouse intraperitoneal infection model, KPC-KP with *mgrB* disruption showed significantly reduced virulence, which likely reflects its potential virulence in humans. This contrasts with the previously reported hypervirulence observed in *Galleria mellonella* (17, 29), suggesting the complexity of mammalian models and their more intricate innate immune responses involving various cell types. The discrepancy in virulence observed between models has also been reported by Russo and MacDonald (30). They found that the outbred mouse infection model effectively differentiated the virulence of hypervirulent *K. pneumoniae* from classical strains, while the *G. mellonella* model showed significant overlap in virulence. Russo and MacDonald suggested that strains with ambiguous pathogenic potential, especially those with antimicrobial resistance that may reduce fitness, should be validated in an outbred murine model. Increased virulence of colistin-resistant strain in *G. mellonella* infection model might be partially due to cross-resistance to antimicrobial peptides naturally produced by *G. mellonella* larvae as part of their innate immunity (31).

On the other hand, multiple bacterial clones can co-colonize the same host during antimicrobial therapy. In our study, following the initial recurrent pneumonia, two colistin-susceptible KPC-KP strains, EDF59 and EDG6, were isolated from sputum. EDF59 and EDG6 display relatively high numbers of SNPs when compared to the initial isolate EDD79, while the subsequent colistin-resistant strain, EDG25, appears to be a direct derivative of EDD79 (Table S1). EDF59 and EDG6 may have co-colonized with EDG25, contributing to the recurrence of pulmonary infection. Thus, the emergence of resistance does not always lead to clinical improvements. The combination therapy of ceftazidime/avibactam and polymyxin B likely eradicated other clones in the patient, but the EDI29 strain, resistant to both ceftazidime/avibactam and colistin, led to significant changes in the clinical condition.

*K. pneumoniae* with *mgrB* inactivation extensively remodels lipid A, the immunogenic part of LPS, through modifications with Ara4N, 2-hydroxymyristate, palmitate, and PEtN (17, 18). LPS, a major pathogen-associated molecular pattern, activates the host innate immune system by binding to TLR-4 on immune cells, triggering an inflammatory response via the NF-κB pathway (32). Modifications in lipid A have been shown to reduce TLR-4 binding, thereby dampening the inflammatory response (13, 15). We found that *mgrB* inactivation in KPC-KP led to a reduced inflammatory response in a mouse pulmonary colonization model. Previous studies have also shown that colistin-resistant strains with pEtN and Ara4N additions elicit attenuated host responses (17, 30). Since a proper inflammatory response is crucial for clearing *K. pneumoniae* infections, the diminished inflammatory response caused by *mgrB* inactivation may facilitate KPC-KP colonization and persistence in the respiratory tract, as observed in our mouse model. This highlights a transition from acute infection to colonization and persistence due to LPS modification in KPC-KP.

It is generally accepted that antibiotic resistance often comes at a cost, leading to reduced bacterial fitness and virulence (33). Our study demonstrates that KPC-KP with *mgrB* disruption shows increased susceptibility to phagocytosis, an attenuated inflammatory response, and reduced virulence *in vivo*. These factors facilitate the transition from acute infection to colonization and persistence in the respiratory tract. This evidence underscores the need to consider antimicrobial resistance and virulence together. Our findings may explain why some patients experience clinical improvement after antimicrobial therapy, despite the emergence of extensive drug resistance and incomplete elimination of pathogenic bacteria.

## Data Availability

Genome sequences of KPC-KP strains have been deposited in the NCBI database under BioProject accession number PRJNA1069333.

## SUPPLEMENTAL MATERIAL

Table S1 aac.01235-24-s0001.docx SNPs among the sequenced KPC-KP isolates.

ASM does not own the copyrights to Supplemental Material that may be linked to, or accessed through, an article. The authors have granted ASM a non-exclusive, world-wide license to publish the Supplemental Material files. Please contact the corresponding author directly for reuse.
