# Supplementary material for: Adaptive evolution of extensive drug resistance and persistence in epidemic ST11 KPC-producing Klebsiella pneumoniae during antimicrobial chemotherapy
Source: Antimicrob Agents Chemother. 2024 Dec 10;69(1):e01235-24. doi: 10.1128/aac.01235-24 (PMC11784014; doi:10.1128/aac.01235-24)
Supplement: Table S1 — SNPs among the sequenced KPC-KP isolates. [file aac.01235-24-s0001.docx]

Table S1. SNPs among the sequenced KPC-KP isolates

| Isolates | Isolates | | | | | | | |
| --- | --- | --- | --- | --- | --- | --- | --- | --- |
|  | EDD79 | EDF59 | EDG6 | EDG25 | EDG92 | EDH1 | EDH17 | EDI29 |
| EDD79 | 0 | 40 | 34 | 13 | 6 | 37 | 8 | 7 |
| EDF59 | 40 | 0 | 7 | 32 | 39 | 5 | 36 | 36 |
| EDG6 | 34 | 7 | 0 | 37 | 36 | 4 | 35 | 35 |
| EDG25 | 13 | 32 | 37 | 0 | 8 | 35 | 7 | 8 |
| EDG92 | 6 | 39 | 36 | 8 | 0 | 38 | 6 | 6 |
| EDH1 | 37 | 5 | 4 | 35 | 38 | 0 | 33 | 31 |
| EDH17 | 8 | 36 | 35 | 7 | 6 | 33 | 0 | 2 |
| EDI29 | 7 | 36 | 35 | 8 | 6 | 31 | 2 | 0 |
